# Genetic Diversity and Phylogenetic Analysis of* Citrus tristeza virus* Isolates from Turkey

**DOI:** 10.1155/2019/7163747

**Published:** 2019-01-01

**Authors:** Gözde Erkiş-Güngör, Bayram Çevik

**Affiliations:** ^1^Applied Sciences University of Isparta, Faculty of Agricultural Sciences and Technologies, Department of Plant Protection, 32260 Isparta, Turkey; ^2^Ministry of Food, Agriculture and Livestock, Antalya Agricultural Quarantine Office, Virology Laboratory, 07260 Antalya, Turkey

## Abstract

The presence of* Citrus tristeza virus *(CTV) in Turkey has been known since the 1960s and the virus was detected in all citrus growing regions of the country. Even though serological and biological characteristics of CTV have been studied since the 1980s, molecular characteristics of CTV isolates have not been studied to date in Turkey. In this study, molecular characteristics of 15 CTV isolates collected from different citrus growing regions of Turkey were determined by amplification, cloning, and sequencing of their major coat protein (CP) genes. The sequence analysis showed that the CP genes were highly conserved among Turkish isolates. However, isolates from different regions showed more genetic variation than isolates from the same region. Turkish isolates were clustered into three phylogenetic groups showing no association with geographical origins, host, or symptoms induced in indicator plants. Phylogenetic analysis of Turkish isolates with isolates from different citrus growing regions of the world including well-characterized type isolates of previously established strain specific groups revealed that some Turkish isolates were closely related to severe quick decline or stem pitting isolates. The results demonstrated that although CTV isolates from Turkey are considered biologically mild, majority of them contain severe components potentially causing quick decline or stem pitting.

## 1. Introduction

Tristeza disease caused by* Citrus tristeza virus* (CTV) has been the most commonly observed viral disease of citrus causing significant economical losses in different countries for more than a century [1, 2]. CTV is a single-stranded positive sense RNA virus belonging to genus* Closterovirus* in* Closteroviridae* family. The virus has flexuous filamentous particles with 2000 nm length and 10-12 nm diameter with about 20 kb genome [1].

The complete genome sequences of CTV isolates from different geographical origins and various biological properties were determined. Genome sequences showed that CTV has the largest plant virus genome ranging from 19226 to 19306 nt and organized into 12 open reading frames (ORF) potentially encoding 17 proteins. Comparison of these genomes also revealed that biologically and geographically different isolates contained a certain degree of sequence variation in different parts of their genomes [3–11]. The p25 gene encoding the major capsid protein (CP) [12] has been used for molecular characterization and typing of CTV isolates around the world [13, 14]. Generally, a correlation between geographical origin and/or biological characteristics of CTV isolates and their CP gene sequences was observed [15–22]. Thus, the CP gene of a large number of CTV isolates with various biological properties from many different citrus growing regions of the world has been sequenced and submitted to GenBank.

As other RNA viruses, CTV isolates do not generally consist of a single unique RNA sequence, but they rather contain of a population of very closely related sequence variants. These variants may result from error made by the RNA-dependent RNA polymerase (RdRp) during replication, recombination in the population, and repeated inoculation in the filed by grafting or insect transmission. Genetic variations associated with insect transmission, different host plants, or host passages were observed in the CP gene of some CTV isolates [23–26]. Thus, sequence analysis of the CP gene not only provides information about genetic diversity and phylogenetic relations of previously uncharacterized isolates but is used for identification of newly identified CTV isolates, strains, or variants.

Symptoms of tristeza disease were first observed in Turkey in the 1960s [27, 28], but the presence of CTV was not demonstrated experimentally until the 1980s [29]. Since the first detection of CTV in eastern Mediterranean region of Turkey [27, 28], it was detected in the Aegean and Black Sea regions. Thus, its presence was confirmed in all citrus growing regions of Turkey. A number of studies were conducted for determination of serological and biological characteristics of CTV isolates found in the eastern Mediterranean region [29–31]. These studies consistently reported that isolates causing only mild vein clearing in Mexican lime were present in Turkey. However, more recent serological and biological characterizations of CTV isolates from citrus growing regions other than the eastern Mediterranean region revealed the existence of MCA13 reactive CTV isolates causing stem pitting on Mexican lime in Turkey [32].

Molecular characterization of the first identified Turkish isolate, Iğdır, by sequence analysis of the CP and the RdRp genes demonstrated that it contained a mixture of mild and severe strains showing homology to stem pitting causing and/or resistance breaking strains of CTV from other citrus growing regions of the world [33]. Although a number of CTV isolates were identified from different citrus hosts and different growing regions, the CP gene sequence of only one isolate from Turkey is known. Therefore, sequencing of more CTV isolates will provide better understanding of genetic diversity of CTV isolates from Turkey. In this study, the CP genes of CTV isolates collected from different citrus growing regions of Turkey were cloned and sequenced. Genetic diversity and phylogenetic relations among isolates from Turkey and other citrus growing regions of the world were determined based on their CP gene sequences.

## 2. Materials and Methods

### 2.1. CTV Isolates

CTV isolates previously collected from different citrus growing regions and identified by ELISA and RT-PCR [32] were used for molecular characterization. Isolates were collected from different citrus species grown in eastern and western Mediterranean regions, the Aegean region, Edremit Gulf of the Marmara region, and the Black Sea region covering all citrus production areas of Turkey. Properties and previously determined biological characteristics [32] of 15 CTV isolates collected from different hosts and representing different citrus growing areas are shown in Table 1. The Iğdır isolate originally obtained from a sweet orange tree grafted on sour orange rootstock in a commercial orchard in Iğdır village of Mersin, Turkey [29, 33], and maintained on Mexican lime to date was also used in this study.

### 2.2. Total RNA Isolation, Amplification, and Cloning of the CP Genes

Total RNA was isolated from bark tissue from young shoots of infected citrus plants. About 100 mg bark tissue was pealed and ground to powder in liquid nitrogen and total RNA was isolated from all samples using One Step RNA Solution (BioBasic, Canada) according to the manufacturer's instructions. Total RNA was dissolved in 50 *μ*l of sterile water and kept at -80°C until used. The CP genes were amplified by two-step reverse transcription polymerase chain reaction (RT-PCR) method. cDNA was synthesized from total RNAs isolated from CTV-infected plants using random hexamer primers and PrimeScript reverse transcriptase (Takara, Japan) as suggested by the manufacture. Then, the CP gene was amplified from the cDNA using* ExTaq* DNA Polymerase (Takara, Japan) with BC24 5′ ATGGACGACGAAACAAAG 3′ and BC25 5′ TCAACGTGTGTTAAATTTCC 3′ primers specific to the CP gene. PCR was conducted in 50 *μ*l PCR mixture containing 1X PCR buffer (50Mm KCl, 10mM Tris HCl 25°C pH 9.0, 1% Triton X-100), 2*μ*l cDNA, 0.5 mM dNTP, 20 pmol BC24 and BC25 primers, and 2.5 units of* ExTaq* DNA polymerase (Takara, Japan). MJ Mini thermal cycler PTC1148 (Bio-Rad, USA) was programmed at 95°C for 3 min for initial denaturation and 40 cycles of 95°C for 30 s for denaturation, 55°C for 30 s for primer annealing, and 72°C for 1 min for primer extension followed by a final extension at 72°C for 5 min. The PCR products were separated 1% agarose gel with 100 bp DNA size makers by electrophoresis, stained with ethidium bromide, and visualized and analyzed by Doc-It system (UVP, England).

CTV CP genes amplified by RT-PCR method were cloned into the pGEM-Teasy plasmid vector (Promega, USA) by the T-A cloning method using T4 DNA ligase.* Escherichia coli* strain JM109 competent cells (Promega, USA) were transformed with ligation mixture of pGEM-Teasy and the CP gene by heat shock procedure as described by the manufacturer. The recombinant colonies containing pGEM-Teasy plasmid vector with the CP gene were selected by blue/white screening on 2X YT medium. Then, bacterial colonies were screened with colony PCR using universal M13R and M13F primers to identify white colonies containing the cloned CP genes. At least two colonies carrying the pGEM-Teasy plasmid with the CP gene were identified for each isolate. Selected colonies were grown in liquid media containing 100 *μ*g mL^−1^ ampicillin, then plasmids were isolated, and the presence of the CP gene was confirmed by* Eco*RI digestion.

### 2.3. Sequencing and Sequence Analysis

The cloned CTV CP genes were sequenced in both directions using universal M13 forward and reverse primers by automated cycle sequencing method. The sequences of the CP genes were assembled and analyzed using Vector NTI Suite program (Invitrogen, USA). Multiple alignments of nucleotide (nt) and amino acid (aa) sequences were performed in AlignX Module of Vector NTI Suite and their percent sequence identity was determined. The refined sequences of CTV CP genes were submitted to the GenBank databases (accession number KU365759 to KU365773). The aligned CP gene sequences were used for construction of phylogenetic trees in MEGA 6 program using neighbor joining algorithm. The constructed phylogenetic tree was tested by a bootstrap analysis with 100 replications to determine the statistical significance of the clustering of CTV isolates with each other or isolates from different citrus growing regions of the world.

Evolutionary analyses of genetic distance and diversity were conducted in MEGA 6 [35]. Average evolutionary distance within and between isolates from different citrus growing regions for Turkish isolates and within and between isolates belonging to previously established groups for all isolates was estimated by the number of base substitutions per site using the nt sequences. Analyses were conducted using the Kimura 2-parameter model [34] and standard error estimate(s) were obtained by a bootstrap procedure (1000 replicates). Both transitional and transversional substitutions were included in the analysis and ambiguous positions were removed for each sequence pair. The average evolutionary diversity for isolates within subpopulations, interpopulations, and entire population as well as of evolutionary differentiation coefficient were estimated by the number of base substitutions per site from the mean diversity calculations [36] using the Kimura 2-parameter model [34]. Standard error was estimated by a bootstrap procedure with 1000 replications. Both transitional and transversional substitutions were included in the analysis and ambiguous positions were removed for each sequence pair.

## 3. Results

### 3.1. Amplification and Cloning of CTV CP Genes

**Figure 1:**
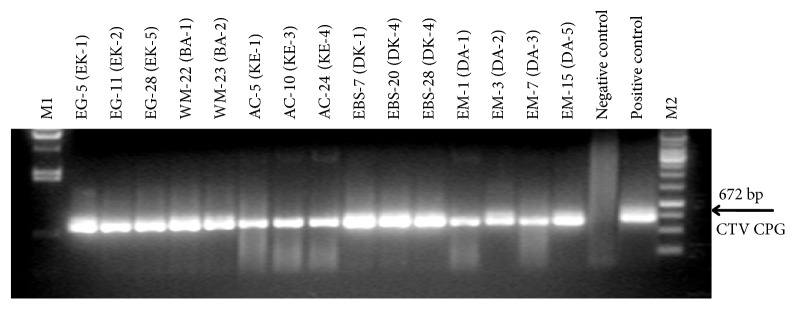
The amplification of 672 bp the CP gene from CTV isolates collected from different citrus growing regions of Turkey. Letters and numbers at the top indicate the names of the isolates. M1 is the* Hind*III digested lambda DNA and M2 is 1 kb DNA ladder. Healthy Madam Vinous sweet orange was used as negative control. Mexican lime infected with previously characterized CTV Iğdır isolate was used as the positive control.

About a 700 bp band corresponding to the CP gene of CTV (672 bp) was amplified from total RNA isolated from Iğdır isolate which was used as positive control and 15 isolates collected from different regions using two-step RT-PCR method. However, no amplification was observed from healthy citrus tissue used as negative control (Figure 1). These results indicated that the CP gene was specifically amplified from 15 different CTV isolates using RT-PCR method. The amplified CP genes were cloned into pGEM-Teasy plasmid and plasmids carrying the CP gene of each isolate were purified from two selected colonies. When the plasmids were cut with* Eco*RI restriction enzyme, majority of clones contained a 3000 bp plasmid and a 700 bp DNA corresponding to the cloned CP gene. However, some clones produced two smaller bands (500 and 200 bp) instead of 700 bp along with the plasmid DNA indicating that they contain an internal* Eco*RI site (data not shown). These results showed that the CP genes of all CTV isolates were cloned and indicated that sequence variations exist among the CP genes of some isolates.

### 3.2. Genetic Variability and Phylogenetic Analysis among Turkish Isolates

Sequence analysis revealed that nt sequences identity in the CP genes of CTV isolates ranged from 91% to 100% (Table 2). The highest sequence identity (100%) was between two Satsuma mandarin isolates: EG28(EK-5) from Edremit Gulf and EBS-20(DK-4) from Black Sea region. Among isolates collected from the same region, the highest similarity (99%) was observed between WM-22(BA-1) and WM-23(BA-2) isolates obtained from Navel orange from Antalya province in western Mediterranean region (Table 2). Based on the nt sequence, EM-1(DA-1) isolate collected from a Yafa orange tree from Cukurova in eastern Mediterranean region was the most different from all other isolates (Table 2). The aa sequences of the CP genes showed 90% to 100% identity (Table 2). While many isolates showed 100% identity, none of them were from the same region. The lowest similarity in aa sequence was observed between isolate EM-1(DA-1) collected from a Shamouti (Yafa) orange tree from eastern Mediterranean region and isolate EM-15(DA-5) collected from a kumquat tree in the same region (Table 2).

**Figure 2:**
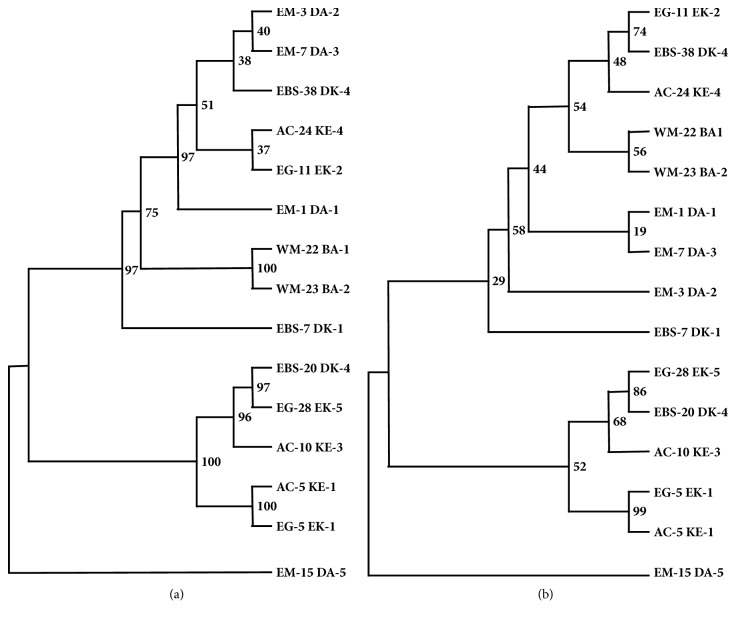
The phylogenetic tree showing genetic relationship among CTV isolates representing different citrus growing regions of Turkey. The phylogenetic tree was constructed with (a) nucleotide and (b) amino acid sequences of the CP gene of Turkish isolates using neighbor joining algorithm in MEGA 6 [35] and tested by a bootstrap analysis with 100 replications. The bootstraps values are shown on nodes of the phylogenetic tree.

Phylogenetic analysis of the nt and aa sequences CTV isolates from different citrus growing regions of Turkey produced very similar phylogenetic trees. Phylogenetic trees showed that isolates were genetically related and divided into three distinct main groups (Figure 2). The branches of the phylogenetic trees were supported by bootstrap values ranging from 38 to 100 for aa and 19 to 99 for nt sequences. The results indicated that branches and clusters were not randomized and that the majority of branches and clusters in the phylogenetic trees were statistically supported. The first group contained nine isolates from five different citrus growing regions of Turkey including eastern [EM-1(DA-1), EM-3(DA-2), and EM-7(DA-3)] and western Mediterranean [WM-22(BA-1) and WM-23(BA-2)], the Aegean cost [AC-24(KE-4)] and Edremit Gulf [EG-11(EK-2)], and eastern Black Sea [EBS-38(DK4)] region. On the other hand, second group contained five isolates from three Satsuma growing regions, the Aegean cost [AC-5(KE-1 and AC-10(KE-3)] and Edremit Gulf [EG-5(EK-1)] and eastern Black Sea EBS-20(DK-3). Unlike these two groups, EM-15(AD-5) isolate collected from a kumquat plant constituted the third group by itself showing that it was the most different among CTV isolates from Turkey. Since two of these groups contained isolates from different citrus growing regions no association with geographical origin of isolates was observed. These isolates did not cause any symptoms on sweet orange and grapefruits and they are considered biologically mild isolates. Therefore, symptoms induced by these isolates on Mexican lime plants (Table 1) were compared with the phylogenetic groups. Since phylogenetic groups contained isolates inducing different symptoms on Mexican lime plants and the third group consisted of only one isolate, no association between phylogenetic groups of Turkish isolates and symptoms on Mexican lime was observed.

Estimation of the average evolutionary divergence of CTV isolates from different citrus growing regions of Turkey based on mean evolutionary distance and diversity is shown in Table 3. Analysis showed that mean evolutionary distance ranged from 0.006±0.003 for isolates from western Mediterranean region to 0.064±0.007 for isolates from eastern Black Sea region with the overall mean of 0.056±0.006 for all different regions. The mean evolutionary distance of isolates between regions ranged from 0.072±0.007 between eastern Mediterranean and Edremit Gulf regions to 0.042±0.005 between eastern and western Mediterranean regions (Table 3). The results showed that the mean evolutionary distance of CTV isolates within and between regions was similar with the exception of isolates from western Mediterranean region.

The genetic diversity analysis revealed that while the mean evolutionary diversity for isolates from the same region (subpopulation) was 0.049±0.005, it was 0.007±0.001 for isolates from different regions (Interpopulation). The mean evolutionary diversity of entire population of CTV isolates was estimated to be 0.056±0.005 and evolutionary differentiation coefficient was 0.126±0.021 (Table 3) suggesting a low genetic variation and a slow evolutionary differentiation amongst CTV isolates in Turkey.

### 3.3. Genetic Diversity and Phylogenetic Analysis Turkish Isolates with World Isolates

The CP gene sequences of CTV isolates from different citrus growing regions of the world with different biological properties and type strains representing previously established strain specific groups were retrieved from gene GenBank databases. Comparison of the CP genes of Turkish CTV isolates with CP genes of international CTV isolates revealed 89-99% identity in their nt sequences indicating that Turkish isolates were similar to the international isolates.

**Figure 3:**
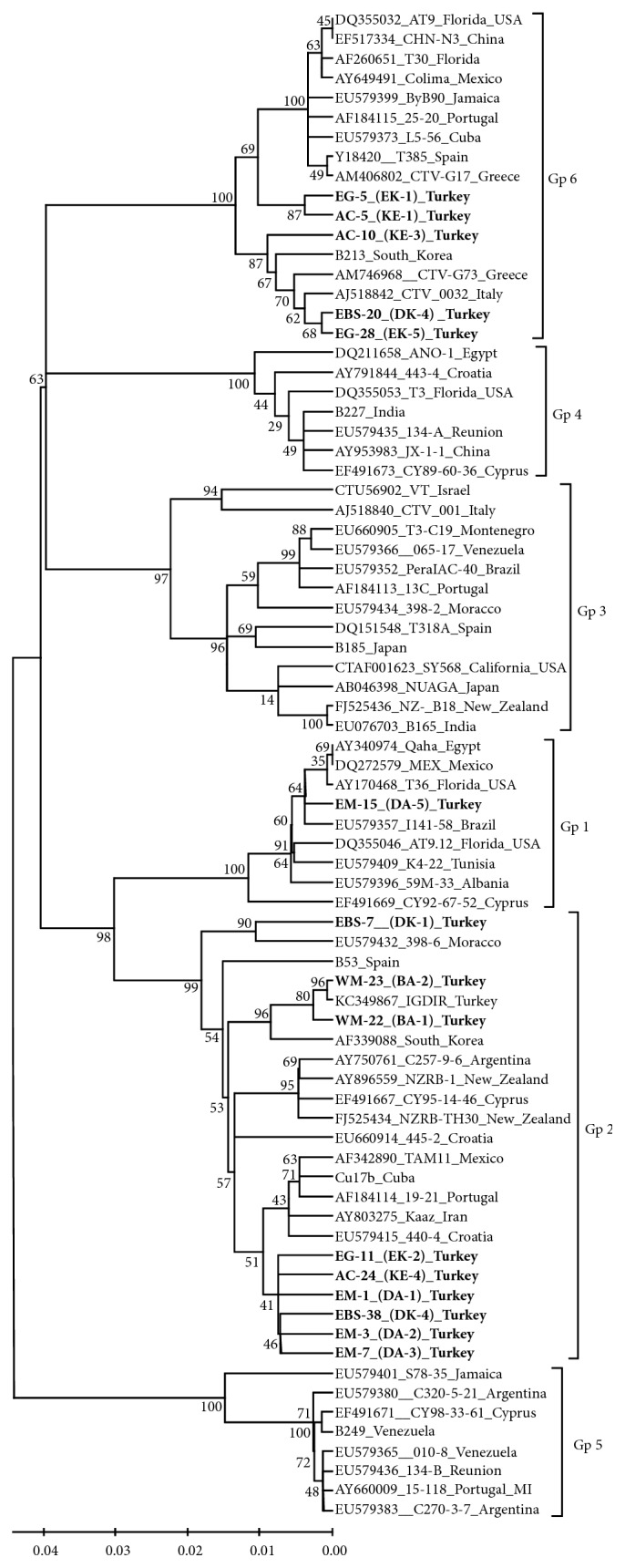
The phylogenetic tree showing genetic relationship between Turkish isolates and isolates from different citrus growing regions of the world including the type isolates of previously established strain specific groups [13, 14]. The phylogenetic tree was constructed with nt sequences of the CP gene of isolates using neighbor joining algorithm in MEGA 6 [35] tested by a bootstrap analysis with 100 replications. The bootstraps values are shown on branches of the phylogenetic tree.

Phylogenetic analysis showed that all CTV isolates were divided into six different groups representing previously established strain specific groups (Figure 3). Turkish isolates were distributed into three of these groups. It was found that majority of Turkish isolates including WM-22(BA-1), WM-23(BA-2), EM-1(DA-1), EM-3(DA-2), EM-7(DA-3), EBS-7(DK-4), EBS-38(DK-4), EG-11(EK-2), and AC-24(KE-4) isolates were clustered with severe CTV isolates such as Clone 19-21 from Portugal, TAM11 from Mexico, and Kaaz from Iran as well as NZRB-1 and NZRB-TH30 resistance braking (RB) isolates from New Zealand (Figure 3). In addition, two type strains of previously identified strain group 2, B53 from Spain and Cu17b from Cuba, were also in this group. Phylogenetic analysis showed that WM-22(BA-1) and WM-23(BA-2) were most related to the first identified CTV isolate, Iğdır, from Turkey and closely grouped with B53 stem pitting (SP) inducing type strain of group 2. However, the other Turkish isolates were tightly clustered with each other and more closely related to Cu17b, another SP inducing type strain representing group 2 (Figure 3). The results suggested that these groups of isolates are closely related to SP causing isolates of CTV and they may contain potentially SP inducing components.

On the other hand, five different isolates from two different regions of Turkey were closely related with previously identified mild isolates of CTV, group 6, from different regions of the world including South Korea, Greece, Italy, Spain, Portugal, Florida, Mexico, and China (Figure 3). While isolates AC10(KE-3), EBS20(DK-4), and EG28(EK-5) were more closely related to one of the type strains of mild group, B213, from South Korean, EG5(EK-1) and AC5(KE-1) were tightly clustered together and more related to T30 the other type strain of the mild CTV isolates. The results showed that these five isolates all from Satsuma mandarin were distantly related to other Turkish isolates and closely related to mild vein clearing causing isolates suggesting that they are most likely mild CTV isolates.

The remaining one isolate EM-15(DA-5) from a kumquat tree was clustered with previously identified QD isolates from Florida, Egypt, Mexico, Brazil, Tunisia, Albania, and Cyprus. This Turkish isolate was the most closely related to 141-58 isolate from Brazil and T36 from Florida, which is the type member of the QD strain group, was also closely related to this isolate. The results indicated that EM-15(DA-5) collected from a kumquat tree in eastern Mediterranean region is a potential QD isolate or it may contain a QD component (Figure 3).

Genetic diversity of CTV isolates from different citrus growing regions of the world including Turkey was estimated as average evolutionary divergence based on mean evolutionary distance and diversity. The results of genetic diversity analyses of isolates from different countries are shown in Table 4. Analysis revealed that the mean evolutionary distance ranged from 0.011±0.002 for isolates from strain group 5 to 0.030±0.004 for isolates from group 3 with the overall mean of 0.089±0.006 for all different groups. The mean evolutionary distance between groups ranged from 0.058±0.011 between groups 1 and 2 to 0.093±0.009 between groups 5 and 6 (Table 4). The results showed that the mean evolutionary distance of CTV isolates within groups was much lower than that of between different groups.

The analysis also revealed that while the mean evolutionary diversity was 0.020±0.001 for isolates from the same strain group (subpopulation), it was 0.049±0.005 for isolates from different strain groups (interpopulation) (Table 4). The mean evolutionary diversity of entire population of CTV isolates was estimated to be 0.070±0.006 and evolutionary differentiation coefficient was 0.7066±0.0183 (Table 4) suggesting low genetic variation but rapid evolutionary differentiation amongst CTV isolates with various biological properties from different regions of the world.

## 4. Discussion

*Citrus tristeza virus* has been present in Turkey since 1963 without causing any major epidemics possibly due to the absence of the most efficient vector, brown citrus aphid (*Toxoptera citricida*), in the country [27, 28]. Biological characterization Turkish isolates by graft inoculation onto indicator plants showed that none of the isolates causes any QD on sweet orange grafted on sour orange or SP on grapefruit or sweet orange. Thus they were all mild isolates causing only vein clearing symptoms on Mexican lime plants [29, 31, 32, 37]. Some of these mild isolates caused vein clearing, leaf cupping, and mild to moderate SP on Mexican lime indicting that they are not typical mild isolates of CTV [32]. Furthermore, sequence analyses of the first identified CTV isolate, Iğdır, which was previously described as biologically a mild isolate, revealed that it actually contains a mixture of mild and severe strains [33]. Therefore, the CP gene sequences of 15 CTV isolates collected from different regions were determined for understanding the genetic diversity and phylogenetic relationships of Turkish isolates with each other and previously characterized international CTV isolates from different citrus growing regions of the world.

Sequence analysis showed that the CP genes of CTV isolates were generally conserved and showed about 10% variation in their nt and aa sequences. Two geographically separated isolates, EG28(EK-5) and EBS20(DK-4), collected from different citrus production regions were genetically the closest isolates with 100% identity in their nt and aa sequences. They were both isolated from Satsuma trees grafted on trifoliate orange rootstock and caused similar symptoms such as vein clearing and slight stem pitting on Mexican lime [32]. In addition, 100% identity was observed between EG-5(EK-1) and AC-5(KE-1) isolates from two different regions as well as among EG-11(EK-2), AC-24(KE4), and EBS-38(DK-4) isolates from three different regions. In spite of the fact that they were geographically separated, they were all isolated from the same host, Satsuma trees grafted on trifoliate orange rootstocks, and caused mild to moderate stem pitting on Mexican lime plants [32].

Overall genetic distance among CTV isolates was similar to genetic distance of isolates within each region except western Mediterranean region, which showed significantly lower genetic distance (0.006±0.003). The lower genetic distance estimated among isolates from this region is likely due to analysis of a fewer number of isolates from this region. Since we have identified only two isolates, no more isolates could be analyzed from this region. Genetic diversity analysis revealed that CTV isolates from the same region were genetically more diverse than the isolates from different regions suggesting that genetic diversity was not associated with geographical origin but may be related to biological characteristics such as host and/or symptom caused in indicator plants [38].

Phylogenetic analysis showed that Turkish isolates were divided into three phylogenetic groups. Since two of these groups contained isolates from different citrus growing regions and different hosts and isolates inducing different symptoms on Mexican lime were in the same group, no association with geographical origin, host, or biological properties of Turkish isolates was observed. Contrary to previous findings showing that phylogenetic grouping of the CP gene is associated with biological properties of CTV isolates from different citrus growing regions [15–17, 19, 39–41], geographical origins and biological properties such as host and/or symptoms induced on an indicator plant Mexican lime had no impact on the phylogenetic grouping. However, further analysis of the CP gene sequences of Turkish isolates with well-characterized international isolates clearly showed that Turkish isolates fall into three of the six previously identified strain groups associated with biological characteristics (see below).

Although the complete genome sequencing is the ideal and most accurate method for identification and strain typing [42], it is not practical and there are only limited numbers of whole genome sequences available from certain regions. Therefore, comparisons and phylogenetic analyses of easily obtained and widely available CP gene sequences have widely been used for molecular characterization and typing of CTV isolate from different geographical origins, various host, and biological properties [13, 14]. Six different strain groups associated with certain biological characteristics regarding symptom induction were determined and group specific probes were designed for differentiation of these groups [13, 39, 43]. These groups have been later confirmed with addition of more isolates from different countries and different biological properties [14]. Due to this association sequence comparisons and the phylogenetic analysis of the CP gene were generally used for typing and characterization of newly identified and/or biologically uncharacterized CTV isolates. Therefore, the CP genes of newly identified Turkish isolate with unknown molecular characteristics and lack of biological data were compared with known international isolates belonging to previously identified strain groups. Phylogenetic analysis of Turkish isolates with international isolates and well-characterized type isolates of previously identified strain groups showed that Turkish isolates were distributed into three different groups. While majority of isolates showed a close phylogenetic relationship with severe isolates causing QD and SP symptoms, some isolates were grouped with mild isolates causing mild vein clearing only on Mexican lime. The phylogenetic analysis showed that nine isolates were associated with group 2 containing well-characterized isolates causing SP on orange and RB isolates able to overcome CTV resistance in* P. trifoliata*. In addition, one isolate from kumquat was closely related with well-known QD isolate, T36, from Florida and other well-characterized QD isolates from different countries. These results suggested that isolates potentially causing SP and QD are present in Turkey. However, none of these isolates induced SP or QD on Madam Vinous orange grafted on sour orange rootstocks in biological indexing [32]. Genetic diversity analysis of the CP genes of isolates within and between phylogenetic groups clearly showed that genetic diversity is much lower in groups consisting of isolates with similar biological characteristics such as symptoms. Furthermore, the existence of association between symptoms and phylogenetic grouping has previously been shown, especially for phylogenetic groups 1, 2, and 6 containing Turkish isolates [14, 44].

Thus, these results are more likely to suggest that Turkish isolate contains mixture of both mild and severe components as we previously demonstrated for one Turkish isolates, Iğdır [33]. Since inoculations for biological indexing were performed by grafting, all components of the mixed isolates are expected to be inoculated. While the CP gene used for phylogenetic analysis is from severe component of isolates, mild component is more dominant in biological indexing assays. Therefore, biologically mild nature of these isolates in the indicator plants may be explained by interaction of different component of the isolate and/or cross protection of severe component by the mild component. CTV isolates containing mixture of mild and severe strains in the same tree were previously reported in different citrus growing regions and both mild and severe components of some of these isolates were separated by aphid transmission and host passage [23, 25] or whole genome sequencing [10, 42]. Similarly, it was previously shown that the first identified Turkish CTV isolates were mixture of at least two different strains and contained sequences similar and phylogenetically related to mild and SP strains were obtained from the same isolate [33]. These suggest that mixed infections with different strains are common and may result in conflicting findings regarding molecular and biological characteristics of CTV isolates. This may be overcome by obtaining monophyletic isolates [42] by separation of different components of CTV isolates by host passage or vector transmission or determination of population structure of isolates by haplotyping with SSCP analysis or sequencing as suggested by [38]. Therefore, more precise determination of association between biological and molecular characteristics of Turkish isolates requires further characterization by indexing in different host, sequencing different parts of the genome or whole genomes.

## Figures and Tables

**Table 1:** Description of CTV isolates used for molecular characterization in this study.

**CTV Isolate** ^**1**^	**Plant/Rootstock**	**Region**	**Symptoms** ^**2**^ ** on Mexican lime** ^**3**^
EG-5(EK-1)	Satsuma/*P. trifoliata*	Edremit Gulf	vc, lc, ch, sp (mild)
EG-11(EK-2)	Satsuma/*P. trifoliata*	Edremit Gulf	vc, sp (modarate)
EG-28(EK-5)	Satsuma/*P. trifoliata*	Edremit Gulf	vc, sp (mild)
WM-22(BA-1)	W. Navel/*C. aurantium*	Western Mediterranean	vc, sp?
WM-23(BA-2)	W. Navel/*C. aurantium*	Western Mediterranean	vc
AC-5(KE-1)	Satsuma/*P. trifoliata*	Aegean Cost	vc, lc, ch, sp (modarate)
AC-10(KE-3)	Satsuma*/P. trifoliata*	Aegean Cost	vc, lc, sp (mild)
AC-24(KE-4)	Satsuma/*P. trifoliata*	Aegean Cost	vc, sp (mild)
EBS-7(DK-1)	Satsuma/*P. trifoliata*	Eastern Black Sea	vc, lc, ch, sp (mild)
EBS-20(DK-4)	Satsuma*/P. trifoliata*	Eastern Black Sea	vc, lc, sp (mild)
EBS-38(DK-4)	Satsuma*/P. trifoliata*	Eastern Black Sea	vc, lc, sp (mild)
EM-1(DA-1)	Shamouti/*C. aurantium*	Eastern Mediterranean	vc
EM-3(DA-2)	Shamouti*/C. aurantium*	Eastern Mediterranean	vc
EM-7(DA-3)	Satsuma/*C. aurantium*	Eastern Mediterranean	vc, sp (mild)
EM-15(DA-5)	Kamquat/*C. aurantium*	Eastern Mediterranean	vc, lc, ch, sp (mild)

^1^EK: Edremit Gulf (EG), BA: Western Mediterranean (WM), KE: Aegean Cost (AC), DK: Eastern Black Sea (EBS), and DA: Eastern Mediterranean (EM).

^2^vc: vein clearing, lc: leaf cupping, ch: chlorosis, and sp: stem pitting.

^3^None of these isolates induced any symptoms on Madam Vinous sweet orange grafted on sour orange, or Madam Vinous sweet orange or grapefruits [32].

**Table 2:** Percent nucleotide (nt) and amino acid (aa) identity of the CP genes of CTV isolates from different citrus growing regions of Turkey.

**Isolates**		**EG-28 (EK-5)**	**AC-10 (KE-3)**	**EG-5 (EK-1)**	**AC-5 (KE-1)**	**EM-15 (DA-5)**	**EBS-7 (DK-1)**	**WM-22 (BA-1)**	**WM-22 (BA-2)**	**EM-1 (DA-1)**	**EM-1 (DA-2)**	**EM-7 (DA-3)**	**EBS-38 (DK-4)**	**EG11 (EK-2)**	**AC-24 (KE-4)**
**EBS-20 (DK-1)**	nt	**100**	99	97	97	93	92	93	93	91	92	92	93	93	92
	aa	**100**	97	96	97	94	96	95	94	91	92	93	95	95	95
**EG-28 (EK-5)**	nt		98	97	97	92	92	93	93	91	92	92	93	93	92
	aa		97	97	97	94	96	96	95	91	92	94	95	95	95
**AC-10 (KE-3)**	nt			97	96	92	92	93	93	91	93	92	93	92	93
	aa			96	96	94	95	94	94	91	93	94	95	95	94
**EG-5 (EK-1)**	nt				99	91	93	92	92	91	91	92	92	93	92
	aa				**100**	94	98	95	95	92	93	95	96	96	96
**AC-5 (KE-1)**	nt					91	93	93	93	91	92	92	93	93	93
	aa					95	98	96	96	92	94	96	97	97	96
**EM-15 (DA-5)**	nt						93	94	94	92	94	93	94	93	93
	aa						95	94	93	**90**	95	95	94	94	94
**EBS-7 (DK-1)**	nt							96	96	95	96	96	96	97	97
	aa							96	96	93	95	96	99	99	98
**WM-22 (BA-1)**	nt								99	96	97	97	97	98	98
	aa								98	94	96	97	99	99	98
**WM-22 (BA-2)**	nt									96	97	97	97	98	98
	aa									94	96	97	99	99	98
**EM-1 (DA-1)**	nt										97	97	97	97	97
	aa										93	95	95	95	95
**EM-3 (DA-2)**	nt											98	99	98	98
	aa											96	97	97	96
**EM-7 (DA-3)**	nt												98	98	98
	aa												99	99	98
**EBS-38 (DK-4)**	nt													99	99
	aa													**100**	**100**
**EG-11 (EK-2)**	nt														99
	aa														**100**

**Table 3:** Estimates of average evolutionary divergence over sequence pairs of the CP genes of CTV isolates from different citrus growing regions of Turkey.

Mean Evolutionary Distance								

Within Regions			Between Regions					

Groups	d	SE	Groups	AC	EG	EBS	EM	WM

AC	0.063	**0.007**	AC	-	**0.005**	**0.006**	**0.007**	**0.007**
EG	0.062	**0.007**	EG	0.045	-	**0.006**	**0.007**	**0.008**
EBS	0.064	**0.007**	EBS	0.052	0.052	-	**0.006**	**0.006**
EM	0.051	**0.006**	EM	0.069	0.072	0.056	-	**0.006**
WM	0.006	**0.003**	WM	0.059	0.060	0.046	0.042	-
Overall	0.056	**0.006**						

Mean Evolutionary Diversity								

				**d**	**SE**			

Within regions				0.049	**0.005**			
Interpopulational				0.007	**0.001**			
Entire Population				0.056	**0.005**			
Coefficient of Evolutionary Differentiation				0.126	**0.021**			

Estimates of standard error (SE) obtained by a bootstrap procedure (1000 replicates) are shown in **bold** in corresponding column. Analyses were conducted using the Kimura 2-parameter model [34]. The analysis involved 15 nucleotide sequences with a total of 672 positions in the final dataset. Evolutionary analyses were conducted in MEGA6 [35] after removing all ambiguous positions for each sequence pair.

**Table 4:** Estimates of the average evolutionary divergence over sequence pairs of the CP genes of CTV isolates from different citrus growing regions of the world belonging to previously established strain groups.

Mean Evolutionary Distance									

Within Groups			Between Groups						

Groups	**d**	**SE**		Group 2	Group 6	Group 4	Group 1	Group 3	Group 5

Group 2	0.026	**0.003**	Group 2		**0.010**	**0.010**	**0.008**	**0.009**	**0.011**
Group 6	0.019	**0.003**	Group 6	0.083		**0.010**	**0.009**	**0.010**	**0.011**
Group 4	0.015	**0.003**	Group 4	0.086	0.080		**0.010**	**0.009**	**0.011**
Group 1	0.021	**0.003**	Group 1	0.058	0.078	0.088		**0.010**	**0.011**
Group 3	0.030	**0.004**	Group 3	0.077	0.081	0.074	0.077		**0.011**
Group 5	0.011	**0.002**	Group 5	0.084	0.093	0.090	0.087	0.090	
Overall	0.089	**0.006**							

Mean Evolutionary Diversity									

				**d**	**SE**				

Within groups				0.020	**0.001**				
Interpopulational				0.049	**0.005**				
Entire Population				0.070	**0.006**				
Coefficient of Evolutionary Differentiation				0.7066	**0.0183**				

Estimates of standard error (SE) obtained by a bootstrap procedure (1000 replicates) are shown in **bold** in corresponding column. Analyses were conducted using the Kimura 2-parameter model [34]. The analysis involved 76 nucleotide sequences with a total of 672 positions in the final dataset. Evolutionary analyses were conducted in MEGA6 [35] after removing all ambiguous positions for each sequence pair.

## Data Availability

The coat protein gene sequence data used to support the findings of this study have been deposited in the GenBank repository under the accession number KU365759.1 to KU365773.1.
